# A machine learning-based predictive model for stem cell therapy outcomes in plastic surgery

**DOI:** 10.3389/fmed.2025.1683758

**Published:** 2025-12-17

**Authors:** Lingang Xu, Ying Lian, Zhen Song, Dongyi Zhang, Hongfeng Zhai

**Affiliations:** Department of Plastic and Cosmetic Surgery, Henan Provincial People’s Hospital, People’s Hospital of Zhengzhou University, Zhengzhou, China

**Keywords:** plastic surgery, stem cell therapy, predictive model, treatment outcome, machine learning

## Abstract

**Objective:**

Stem cell therapy has emerged as a promising approach in plastic surgery, yet its efficacy varies markedly among individuals and lacks reliable predictive assessment tools. This study aimed to construct and validate a predictive model for assessing the therapeutic efficacy of stem cell therapy in plastic surgery by identifying key influencing factors through clinical data analysis and machine learning.

**Methods:**

Patients who underwent stem cell therapy in the Department of Plastic Surgery from June 2021 to July 2024 were retrospectively included and randomly divided into a training set and a validation set at a 7:3 ratio. Baseline clinical data were collected, and independent influencing factors were screened via univariate analysis, followed by multivariate logistic regression and LASSO feature selection in the training set. Three machine learning models—random forest (RF), support vector machine (SVM), and K-nearest neighbors (KNN)—were constructed using Python 3.8.5 and the scikit-learn library, followed by performance validation in the validation set.

**Results:**

A total of 620 patients who underwent stem cell therapy were included. In the training set (*n* = 434), 262 cases (60.37%) showed effective treatment outcomes, while 112 cases (60.23%) were effective in the validation set (*n* = 186). Multivariate logistic regression revealed that age, disease duration, diabetes history, and cell passage number were independent risk factors for therapeutic efficacy (all *P* < 0.05), whereas baseline skin score, stem cell dosage, and injection frequency were independent protective factors (all *P* < 0.05). The AUC values of the RF, SVM, and KNN models were 0.798, 0.770 and 0.723 in training set, and 0.787, 0.761 and 0.708 in validation set, respectively, with the RF model demonstrating superior performance.

**Conclusion:**

The machine learning-based predictive model for stem cell therapy efficacy in plastic surgery, constructed through clinical data analysis, exhibits moderate predictive accuracy and may serve as a reference for clinical personalized treatment.

## Introduction

Stem cell therapy has been widely applied in plastic surgery for tissue regeneration, demonstrating significant efficacy in treating conditions such as alopecia areata, skin aging, and scar repair (1). From a cellular biological perspective, stem cells exhibit multipotent differentiation potential and paracrine effects, promoting angiogenesis, modulating the immune microenvironment, and inducing tissue regeneration, thereby improving the survival microenvironment of fat grafts and remodeling collagen fiber structure (2, 3). However, substantial interindividual variability in therapeutic outcomes has been observed clinically. For instance, in fat transplantation, clinical data indicate a survival rate ranging from 30% to 80%, with some patients requiring repeated surgical interventions (2–3 times) due to suboptimal graft retention. Such marked heterogeneity in treatment efficacy significantly increases both financial burden and psychological distress for patients (4). Currently, the assessment of stem cell therapy efficacy in plastic surgery primarily relies on clinicians’ empirical judgment and postoperative imaging examinations (ultrasonography or magnetic resonance imaging for volumetric measurement of transplanted tissues), both of which have inherent limitations. Thus, identifying novel predictive markers is imperative (5, 6). Most existing predictive models in stem cell-based plastic surgery focus on single indications or rely on traditional regression. These models lack generalizability to multiple conditions and practical tools. In contrast, our study integrates alopecia areata, skin aging, and scar repair—three common indications—uses Least Absolute Shrinkage and Selection Operator (LASSO) for robust feature selection and machine learning [random forest (RF), support vector machine (SVM), and K-nearest neighbors (KNN)] for modeling, addressing the unmet need for multi-indication predictive tools in clinical practice.

Recent advancements in clinical data analytics and machine learning offer a promising solution to this challenge. By integrating clinical data, cellular biological characteristics, and imaging information, machine learning models can establish a multidimensional predictive framework (7–9). Accordingly, this study aims to screen statistically significant variables from univariate analysis, identify independent influencing factors via multivariate logistic regression, and ultimately integrate key variables to construct and validate interpretable predictive models—including random forest, support vector machine, and K-nearest neighbor algorithms—to achieve individualized efficacy prediction for stem cell therapy, thereby supporting clinical decision-making in treatment optimization and patient stratification.

## Materials and methods

### Study subjects

This retrospective study enrolled 620 patients who underwent stem cell therapy in the Department of Plastic Surgery of our hospital from June 2021 to July 2024, including 182 cases (29.4%) of alopecia areata, 265 cases (42.7%) of skin aging, and 173 cases (27.9%) of scar repair. To enhance statistical power, develop a multi-indication predictive tool applicable to common clinical scenarios, and verify the generalizability of key predictors, we intentionally pooled these three indications and subsequently conducted subgroup analyses to confirm the consistency of predictive factors across different conditions. Inclusion criteria: (1) age ≥ 18 years, (2) treatment with autologous adipose-derived stem cells (ADSCs) or mesenchymal stem cells (MSCs), (3) complete clinical records. Exclusion criteria: (1) severe systemic comorbidities, (2) major psychiatric disorders, (3) loss to follow-up. The patients were randomly divided into a training set (*n* = 434) and a validation set (*n* = 186) at a ratio of 7:3. All patients gave informed consent and voluntarily participated in this study, and the study was approved by the hospital’s ethics committee.

### Data collection

Demographic characteristics [age, gender, body mass index (BMI), smoking and drinking history], clinical features (disease duration, hypertension, diabetes, comorbidities, and pre-treatment skin score), stem cell treatment parameters (cell type, source, dose, concentration, passage number, culture duration, injection frequency, and number of injection sites), and 6-month follow-up efficacy outcomes were collected. Pre-treatment skin score was assessed using the Modified Skin Quality Scoring System (MSQS) (10), a validated 100-point scale evaluating elasticity, hydration, texture, and vascularity via standardized clinical examination and imaging. Higher scores (≥ 65) indicated better baseline skin health, as previously correlated with stem cell engraftment success.

### Stem cell therapy protocol

#### Cell source and isolation

ADSCs: Harvested from autologous abdominal or thigh adipose tissue, digested with 0.1% collagenase (37 °C, 60 min), and centrifuged at 1,200 rpm for 10 min. Pelleted cells were cultured in DMEM/F12 medium supplemented with 10% fetal bovine serum.

MSCs: Derived from autologous bone marrow (iliac crest aspiration) or umbilical cord tissue (donated by cesarean-section mothers). Bone marrow mononuclear cells were isolated via density gradient centrifugation, while cord tissue was minced and enzymatically dissociated. Cells were cultured in α-MEM medium.

#### Treatment protocol

Alopecia areata: Intradermal injection of 0.1 mL stem cell suspension (20 × 10^6^ cells/mL) per cm^2^ of alopecic area, monthly for 3–4 sessions.

Skin aging: Multipoint subcutaneous injections (5–8 sites per facial side, 0.2–0.3 mL/site at 25 × 10^6^ cells/mL), bimonthly for 2–3 sessions.

Scar repair: Intralesional/perilesional injections adjusted by scar size (total 2–5 mL/session at 15 × 10^6^ cells/mL), monthly for 4–6 sessions.

### Efficacy evaluation

Patients were classified per Stem Cell Therapy Efficacy Evaluation guidelines (11). “Effective” treatment was defined as ≥ 50% improvement in target regions, operationalized as: for alopecia areata, ≥ 50% increase in hair density per cm^2^ assessed by standardized photographic analysis; for skin aging, ≥ 50% reduction in wrinkle depth measured by the Fitzpatrick Wrinkle Assessment Scale or improvement in skin texture score; for scar repair, ≥ 50% reduction in volume via ultrasonography or improvement in pliability using the Vancouver Scar Scale. This threshold was established by consensus among plastic surgery societies for clinically meaningful outcomes. “Ineffective” denoted < 50% improvement, validated against patient-reported satisfaction scores and blinded clinician assessments. Efficacy was assessed by two independent plastic surgeons blinded to patients’ baseline data, and inter-rater reliability was measured with Cohen’s kappa = 0.82 (good agreement), ensuring the objectivity of efficacy assessment.

### Statistical analysis

SPSS 26.0, R 4.2.3 and Python 3.8.5 were used for statistical analyses. Normally distributed continuous variables were expressed as mean ± SD and compared via *t*-tests, categorical data were presented as counts (percentages) and analyzed by χ^2^ tests. In the training set, variables with *P* < 0.05 in univariate analysis were included in multivariate logistic regression to identify independent predictors. LASSO regression (implemented via the “glmnet” package in R) was used for feature selection in the training set to identify optimal predictors, which can mitigate collinearity and overfitting. The lambda value was determined by 5-fold cross-validation (lambda.min). Only the training set was used for variable selection and model training, while the validation set was reserved for independent performance testing to avoid data leakage. Collinearity of the final selected predictors was verified using variance inflation factor (VIF) (all VIF < 5). Using Python 3.8.5 (scikit-learn library), RF, SVM, and KNN models were constructed. For RF, feature scaling was not applied as it is tree-based and insensitive to feature magnitudes; however, for SVM and KNN, features were not scaled, which may disadvantage their performance relative to RF due to sensitivity to feature scales. Five-fold cross-validation optimized hyperparameters. Model performance was evaluated by accuracy, precision, recall, F1-score, and area under the receiver operating characteristic (ROC) curve (AUC), with the highest AUC and F1-score determining the optimal model. A calibration curve was plotted and evaluated using the Hosmer–Lemeshow goodness–of–fit test. Decision curve analysis (DCA) was used to evaluate the clinical application value of the optimal model by calculating the net benefit at different threshold probabilities. Hyperparameter tuning was performed via 5-fold cross-validation with grid search: for RF, parameters included n_estimators (100–300) and max_depth (5–10), with optimal values of 200 and 8, respectively; for SVM, kernel type (linear/rbf) and C (0.1–10) were tuned, with linear kernel and *C* = 1 selected. No missing values were present as inclusion criteria required complete clinical records. Class imbalance was not observed (effective rate: 60.37% in training set, 60.23% in validation set), so no SMOTE or class weighting was applied. A *P*-value < 0.05 was considered statistically significant.

## Results

### Baseline characteristics of training and validation sets

A total of 620 patients who underwent stem cell therapy were enrolled. There were 262 (60.37%) effective cases in the training set (*n* = 434) and 112 (60.23%) effective cases in the validation set (*n* = 186). No significant intergroup differences in baseline characteristics were observed (*P* > 0.05) (Table 1).

**TABLE 1:** Comparison of baseline characteristics between training and validation sets.

Indicators	Training set (*n* = 434)	Validation set (*n* = 186)	*t*/χ ^2^	*P*
Age (years)	41.05 ± 10.67	41.89 ± 10.93	0.892	0.373
BMI (kg/m^2^)	23.04 ± 3.25	23.18 ± 3.33	0.488	0.626
Sex (male/female)	167 (38.48)/267 (61.52)	71 (38.17)/115 (61.83)	0.005	0.943
Disease duration (months)	15.89 ± 6.61	16.11 ± 6.74	0.378	0.706
Disease type (alopecia/skin aging/scar repair)	127 (29.26%)/185 (42.63%)/122 (28.11%)	55 (29.57%)/80 (43.01%)/51 (27.42%)	0.126	0.891
Drinking history (yes/no)	75 (17.28)/359 (82.72)	40 (21.51)/146 (78.49)	1.538	0.215
Smoking history (yes/no)	83 (19.12)/351 (80.88)	38 (20.43)/152 (79.57)	0.065	0.799
Hypertension (yes/no)	92 (21.20)/342 (78.80)	41 (22.04)/145 (77.96)	0.055	0.814
Diabetes (yes/no)	51 (11.75)/383 (88.25)	23 (12.37)/163 (87.63)	0.047	0.829
Comorbidity (yes/no)	105 (24.20)/329 (75.80)	53 (28.49)/133 (71.51)	1.268	0.260
Pre-treatment skin score	63.14 ± 10.05	62.89 ± 10.85	0.277	0.782
Stem cell type (ADSC/MSC)	220 (50.69)/214 (49.31)	102 (54.84)/84 (45.16)	0.897	0.344
Stem cell source (autologous/allogeneic)	305 (70.28)/129 (29.72)	125 (67.20)/61 (32.80)	0.578	0.447
Stem cell dose (×10^6^)	6.87 ± 2.51	6.64 ± 2.49	1.048	0.295
Stem cell concentration (×10^6^/ml)	22.18 ± 5.73	21.75 ± 5.91	0.848	0.397
Passage number (≤ 3/ > 3)	356 (82.03)/78 (17.97)	152 (81.72%)/34 (18.28%)	0.021	0.884
Culture duration (days)	7.36 ± 1.48	7.44 ± 1.50	0.614	0.539
Number of injections	2.88 ± 0.74	2.81 ± 0.77	1.066	0.287
Number of injection sites	4.47 ± 1.20	4.52 ± 1.26	0.468	0.640

### Univariate analysis of factors influencing the efficacy of stem cell therapy

Univariate analysis revealed that in the training set, significant differences were observed between the effective and ineffective groups regarding patient age, disease duration, history of diabetes, pre-treatment skin score, stem cell dosage, passage number, and injection frequency (all *P* < 0.05) (Table 2).

**TABLE 2:** Univariate analysis of factors influencing the efficacy of stem cell therapy.

Indicators	Effective group (*n* = 262)	Ineffective group (*n* = 172)	*t*/χ^2^	*P*
Age (years)	39.22 ± 10.15	43.86 ± 10.93	4.518	0.001
BMI (kg/m^2^)	22.95 ± 3.18	23.28 ± 3.36	1.034	0.302
Sex (male/female)	95 (36.26)/167 (63.74)	72 (41.86)/100 (58.14)	1.376	0.241
Disease duration (months)	13.90 ± 6.58	18.05 ± 6.77	6.354	0.001
Disease type (alopecia/skin aging/scar repair)	76 (29.01%)/112 (42.75%)/74 (28.24%)	51 (29.65%)/73 (42.44%)/48 (27.91%)	0.298	0.763
Drinking history (yes/no)	42 (16.03)/220 (83.97)	33 (19.19)/139 (80.81)	0.723	0.395
Smoking history (yes/no)	52 (19.85)/210 (80.15)	31 (18.02)/141 (81.98)	0.223	0.637
Hypertension (yes/no)	51 (19.47)/211 (80.53)	41 (23.84)/131 (76.16)	1.188	0.276
Diabetes (yes/no)	20 (7.63)/242 (92.37)	31 (18.02)/141 (81.98)	7.172	0.001
Comorbidity (yes/no)	62 (23.66)/200 (76.37)	43 (25.00)/129 (75.00)	0.101	0.751
Pre-treatment skin score	65.08 ± 10.14	60.92 ± 10.01	4.202	0.001
Stem cell type (ADSC/MSC)	139 (53.05)/123 (46.95)	81 (47.09)/91 (52.91)	1.476	0.224
Stem cell source (autologous/allogeneic)	189 (72.14)/73 (27.86)	116 (67.44)/56 (32.56)	1.096	0.295
Stem cell dose (×10^6^)	7.49 ± 2.62	6.38 ± 2.38	4.475	0.001
Stem cell concentration (×10^6^/ml)	22.26 ± 5.79	21.98 ± 5.84	0.491	0.624
Passage number (≤ 3/ > 3)	229 (87.40)/33 (12.60)	127 (73.84)/45 (26.16)	12.965	0.001
Culture duration (days)	7.52 ± 1.51	7.31 ± 1.39	1.462	0.145
Number of injections	3.05 ± 0.81	2.79 ± 0.72	3.416	0.001
Number of injection sites	4.52 ± 1.35	4.40 ± 1.18	0.951	0.342

### Multivariate logistic regression analysis of factors influencing the efficacy of stem cell therapy

Using the efficacy of stem cell therapy (1 = ineffective, 0 = effective) as the dependent variable, variables that demonstrated statistical significance in the univariate analysis were included as independent variables in the multivariate logistic regression analysis (Supplementary Table 1). LASSO regression in the training set selected 7 predictors (age, disease duration, diabetes history, pre-treatment skin score, stem cell dose, passage number, injection frequency) with lambda.min = 0.023. The results indicated that age, disease duration, history of diabetes, and passage number were independent risk factors for treatment efficacy (all *P* < 0.05), whereas pre-treatment skin score, stem cell dose, and number of injections were independent protective factors (all *P* < 0.05) (Table 3). VIF values for these predictors ranged from 1.12 to 3.87 (all < 5), confirming no collinearity.

**TABLE 3:** Multivariate logistic regression analysis of factors influencing the efficacy of stem cell therapy.

Factor	β	SE	Wald	*P*	OR	95% CI
Age	0.052	0.012	19.616	0.001	1.053	1.029–1.077
Disease duration	0.096	0.018	27.492	0.001	1.101	1.062–1.142
History of diabetes	0.825	0.351	5.543	0.019	2.283	1.148–4.538
Pre-treatment skin score	−0.045	0.012	15.428	0.001	0.956	0.934–0.978
Stem cell dose	−0.201	0.047	18.244	0.001	0.818	0.745–0.897
Passage number	0.919	0.292	9.942	0.002	2.508	1.146–4.441
Number of injections	−0.545	0.152	12.920	0.001	0.580	0.431–0.781

### Subgroup analysis by disease type

Multivariate logistic regression analyses across disease-specific subgroups demonstrated high consistency with the overall model. The core predictive factors remained directionally aligned (risk or protective) and statistically significant in all subgroups, with no contradictory associations observed (Table 4).

**TABLE 4:** Multivariate logistic regression analysis of factors influencing stem cell therapy efficacy across disease subgroups.

Disease type	Factor	OR (95% CI)	*P*-value
Alopecia areata (*n* = 182)	Age	1.048 (1.012–1.085)	0.009
	Disease duration	1.085 (1.023–1.151)	0.007
	Stem cell dose	0.792 (0.710–0.883)	0.002
	Injection frequency	0.587 (0.452–0.763)	< 0.001
	Passage number	2.356 (1.210–4.586)	0.012
	Diabetes history	2.215 (1.058–4.639)	0.035
Skin aging (*n* = 265)	Pre-treatment skin score	0.945 (0.916–0.975)	< 0.001
	Stem cell dose	0.805 (0.728–0.890)	< 0.001
	Age	1.051 (1.018–1.085)	0.002
	Passage number	2.412 (1.288–4.513)	0.006
	Injection frequency	0.561 (0.445–0.707)	< 0.001
Scar repair (*n* = 173)	Diabetes history	2.298 (1.175–4.509)	0.016
	Passage number	2.462 (1.280–4.734)	0.007
	Disease duration	1.090 (1.025–1.159)	0.006
	Injection frequency	0.593 (0.440–0.799)	0.001

### Predictive performance of machine learning models in training and validation sets

**FIGURE 1:**
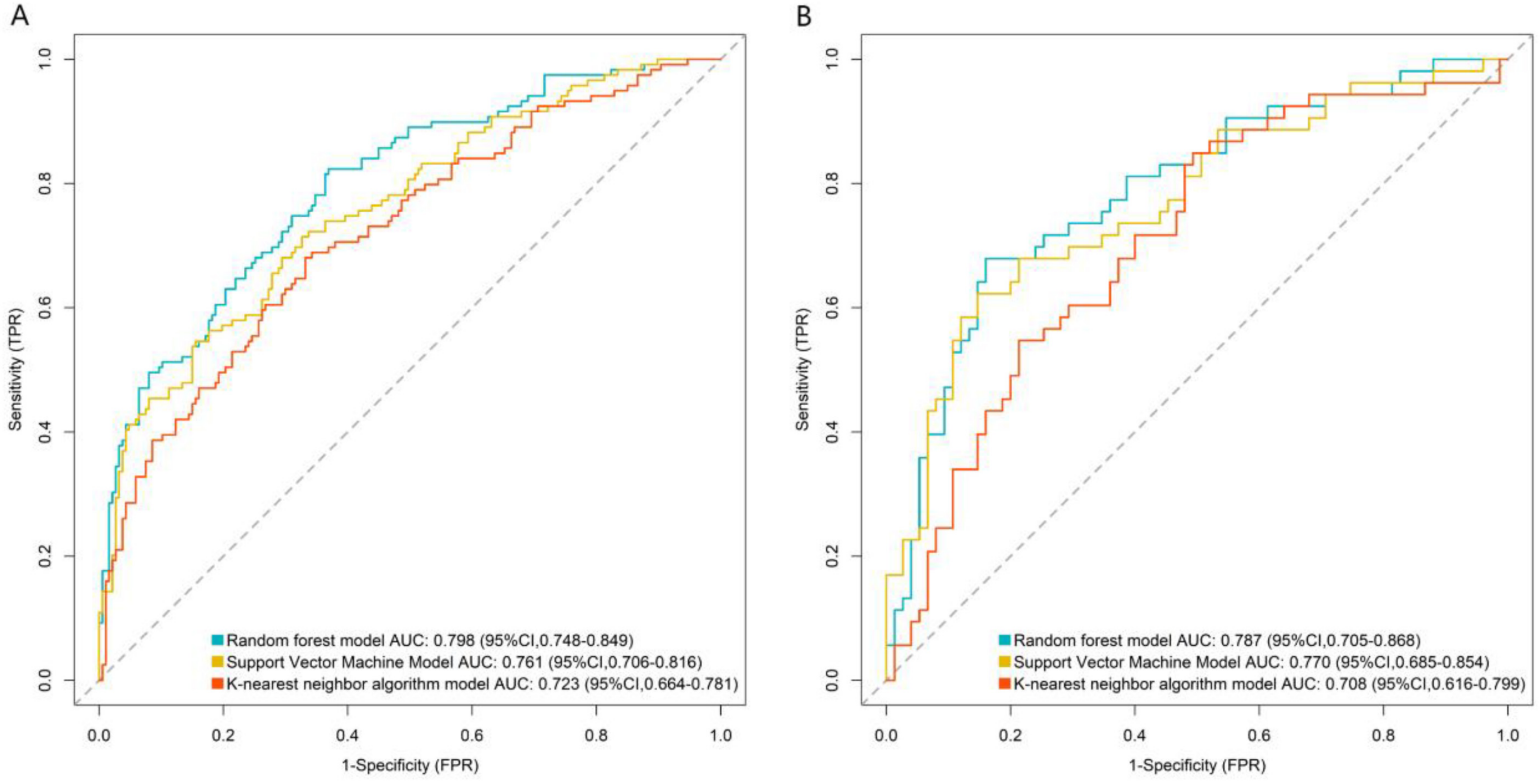
Receiver operating characteristic curves of machine learning models in the training set **(A)** and the validation set **(B)**.

The RF, SVM, and KNN models were applied to predict outcomes in both the training and validation sets. The evaluation metrics for each model were calculated, and the model with the highest AUC and F1-score was selected as the optimal model for this study, which was determined to be the RF model (Table 5 and Figure 1).

**TABLE 5:** Predictive performance of models in the training set and the validation set.

Group	Model	Precision	Accuracy	Recall	F1 value	AUC	Specificity	Sensitivity
Training set	Random forest model	0.816	0.823	0.842	0.822 (0.781–0.863)	0.798 (0.748–0.849)	0.887	0.851
	Support vector machine model	0.785	0.813	0.835	0.795 (0.729–0.861)	0.770 (0.685–0.854)	0.844	0.819
	K-nearest neighbor algorithm model	0.754	0.766	0.771	0.768 (0.721–0.815)	0.723 (0.664–0.781)	0.812	0.795
Validation set	Random forest model	0.801	0.817	0.863	0.816 (0.754–0.878)	0.787 (0.705–0.868)	0.863	0.847
	Support vector machine model	0.782	0.801	0.814	0.783 (0.738–0.828)	0.761 (0.706–0.816)	0.854	0.824
	K-nearest neighbor algorithm model	0.743	0.752	0.789	0.774 (0.703–0.845)	0.708 (0.616–0.799)	0.809	0.778

### Calibration and decision curve analysis of machine learning models

**FIGURE 2:**
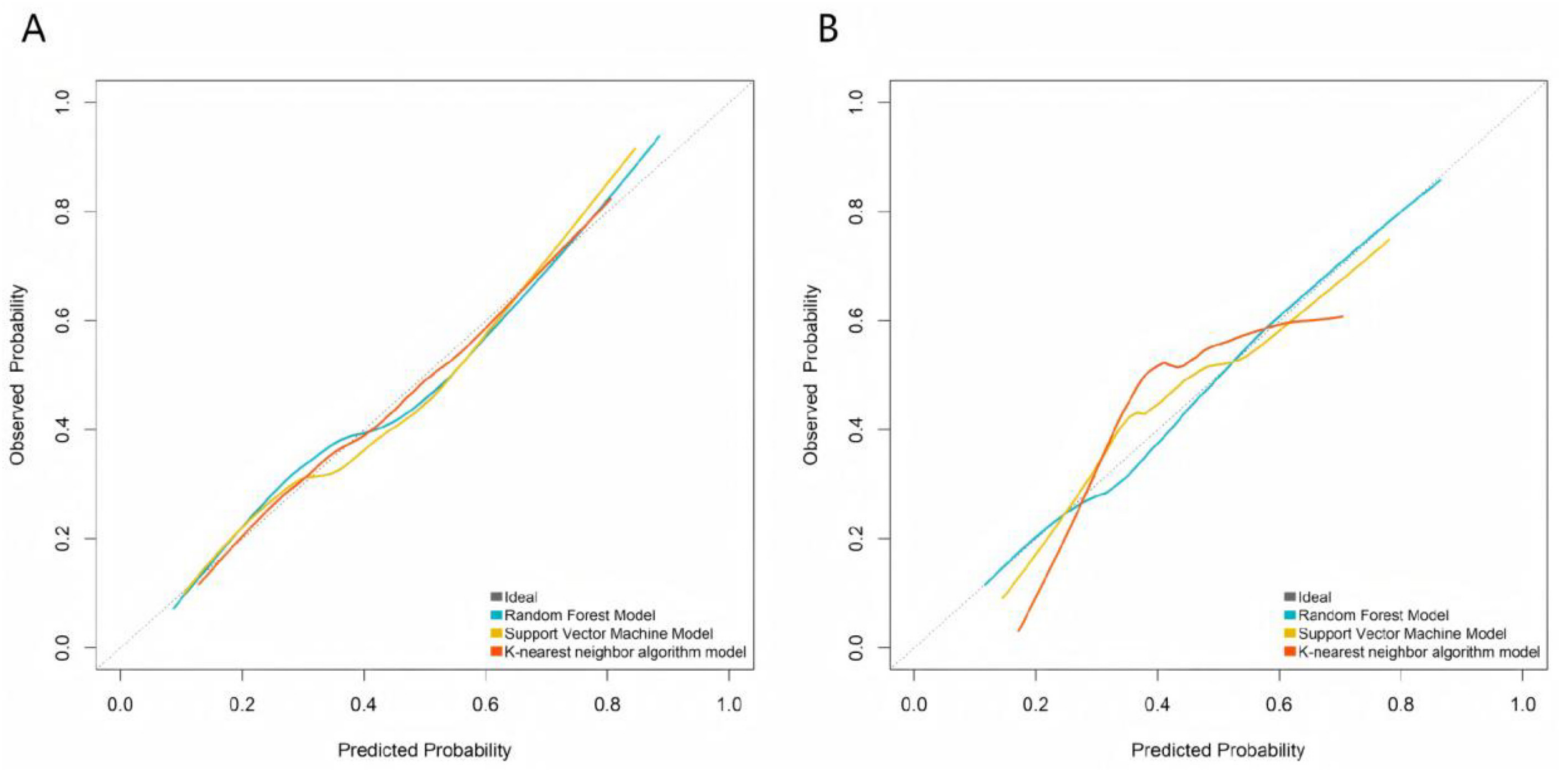
Calibration curves of machine learning models in the training set **(A)** and the validation set **(B)**.

**FIGURE 3:**
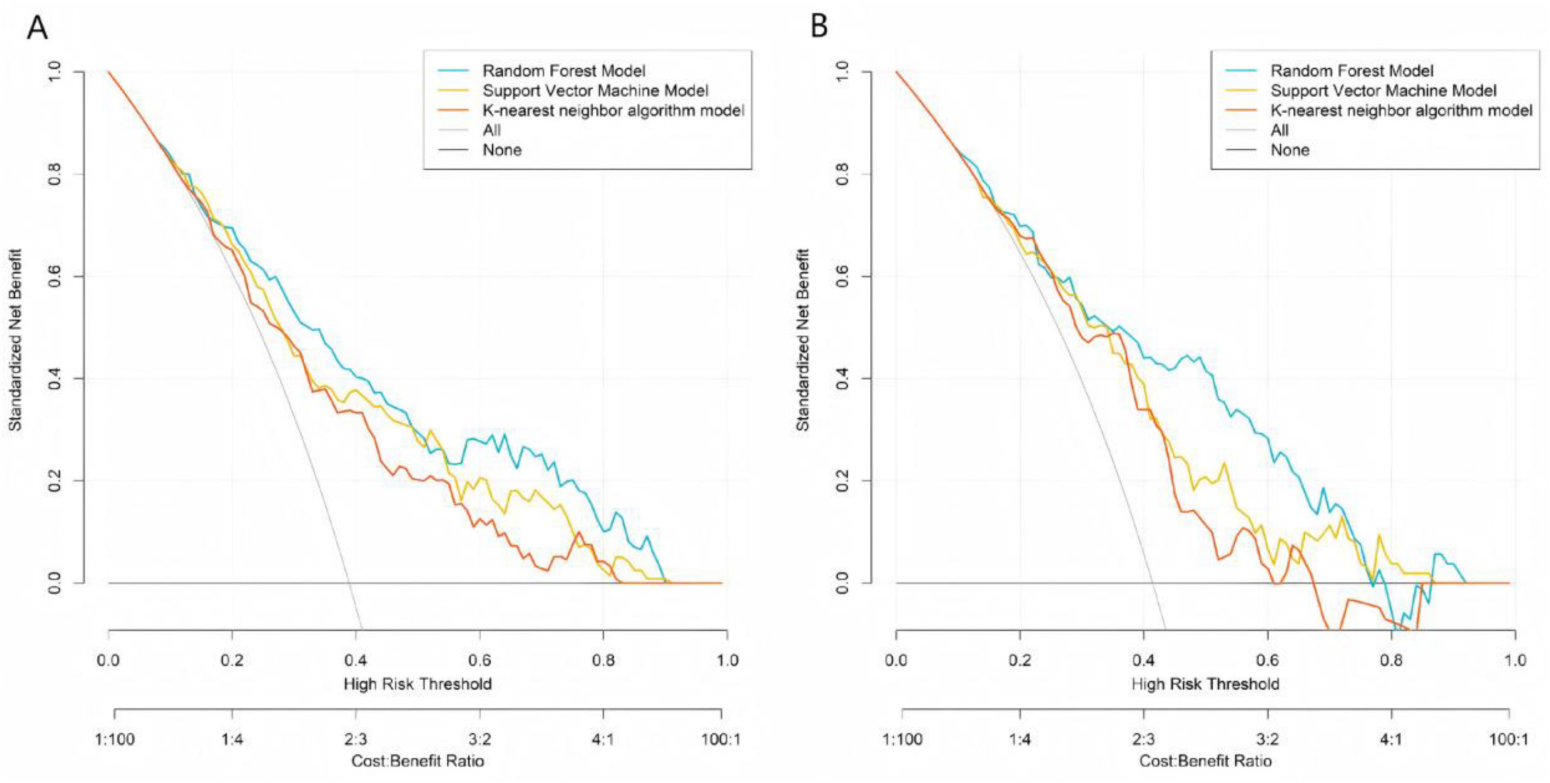
Decision curve analysis of machine learning models in the training set **(A)** and the validation set **(B)**.

Calibration curves demonstrated that the predicted probabilities of treatment ineffectiveness from the RF, SVM, and KNN models showed reasonable agreement with the actual observed incidence rates (Figure 2). The RF model’s calibration curve most closely approximated the “ideal” diagonal line in both the training and validation sets. The more pronounced deviations of the SVM and KNN curves from the ideal line, particularly in the validation set, indicate that the RF model provides superior accuracy in estimating the risk of treatment ineffectiveness and possesses better generalization capability. DCA showed that across a wide range of risk thresholds, the standardized net benefits of all three models significantly exceeded those of the extreme strategies of “treating all” or “treating none.” The random forest demonstrated a particularly prominent advantage: in the training set, at the clinically common risk threshold of 0.30 (where a predicted inefficacy risk of 30% may prompt treatment intensification, such as increasing injection frequency or adding adjunct therapies like PRP), its net benefit was significantly higher than those of the SVM and KNN models, and it maintained the highest net benefit throughout the core clinical decision range (risk thresholds 0.20–0.60). Similarly, in the validation set, the RF model’s net benefit at the 0.30 threshold and across the core decision interval was significantly superior to the other models, robustly demonstrating its practical value in guiding individualized clinical treatment decisions (Figure 3).

### Construction of the stem cell therapy efficacy prediction model

The importance scores of factors influencing stem cell therapy efficacy were calculated based on the RF model, yielding the following descending order of significance: age, disease duration, stem cell dosage, passage number, diabetes history, number of injections, and pre-treatment skin score (Supplementary Figures 1, 2).

**FIGURE 4:**
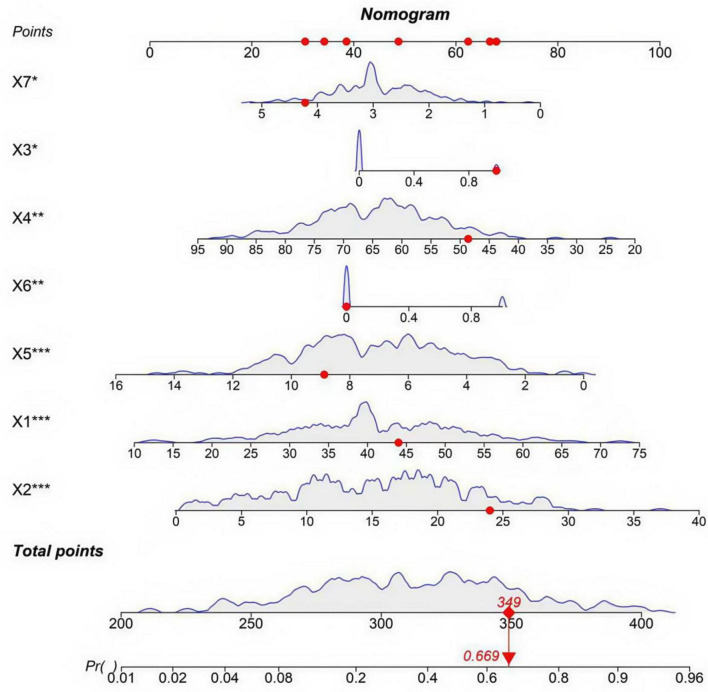
Nomogram for predicting the risk of stem cell therapy ineffectiveness. X1: age (years); X2: disease duration (months); X3: history of diabetes (0 = No, 1 = Yes); X4: pre-treatment skin score; X5: stem cell dose (×10^6^); X6: passage number (0 ≤ 3, 1 ≥ 3); X7: number of injections. The symbols indicate the statistical significance of independent predictors based on multivariate logistic regression: *P < 0.05; **P < 0.01; ***P < 0.001.

To facilitate clinical application, a nomogram was developed based on the random forest incorporating the seven independent predictors (Figure 4). Each predictor is assigned a score on the points scale; the sum of all scores corresponds to the total points, which can be converted to the predicted probability of treatment ineffectiveness. For risk stratification, we propose expert-driven cutoffs: total points corresponding to ≥ 60% probability define high-risk, and < 30% define low-risk, based on clinical consensus for decision-making (12).

## Discussion

This study analyzed clinical data from 620 patients undergoing stem cell therapy in plastic surgery and identified age, disease duration, history of diabetes, passage number, pre-treatment skin score, stem cell dose, and injection frequency were identified as independent protective factors through multivariate logistic regression analysis. Among the predictive models constructed based on RF, SVM, and KNN algorithms, the RF model demonstrated the best performance, with AUC values of 0.798 (training set) and 0.787 (validation set), significantly outperforming the other models. These findings indicate that the machine learning-based predictive model for stem cell therapy efficacy has substantial clinical utility, providing a reliable tool for early identification of high-risk patients.

The multivariate analysis revealed key drivers of individual variability in therapeutic efficacy, with each factor’s mechanism aligning well with clinical observations. Age, as an independent risk factor (OR = 1.053), indicates that each additional year of age increases the odds of treatment ineffectiveness by 5.3%, consistent with age-related degenerative changes in stem cell functionality (13). Age correlates with age-related declines in stem cell function—senescent microenvironments reduce VEGF secretion (14–16), weakening tissue repair, which aligns with our finding that older patients have lower efficacy. Disease duration exacerbates local fibrosis, hindering stem cell engraftment, consistent with our observation of poorer outcomes in patients with > 18 months of disease. This was comparable to Margiana et al.’s fat graft model, where disease chronicity also emerged as a key predictor (17–19). A history of diabetes significantly increases treatment failure risk (OR = 2.283) through multiple mechanisms: hyperglycemia-induced non-enzymatic glycation damages vascular endothelial cells, reducing local perfusion and limiting oxygen and nutrient supply essential for stem cell survival (20, 21). Additionally, diabetes-associated oxidative stress activates the NF-κB pathway, promoting pro-inflammatory cytokine release and suppressing stem cell paracrine effects and regenerative capacity (22, 23). High passage number (> 3) (OR = 2.508) aligns with the Hayflick limit theory of cellular senescence, where excessive passaging leads to telomere shortening, DNA damage accumulation, and impaired proliferation and secretory function; studies show that VEGF secretion decreases by > 40% in passage-4 stem cells compared to passage-2 cells (24, 25).

In contrast, pre-treatment skin score (OR = 0.956), stem cell dose (OR = 0.818), and injection frequency (OR = 0.580) exhibited protective effects, reflecting a synergistic “baseline status–treatment strategy” relationship. A higher skin score (> 65) indicates intact collagen structure and rich dermal papillary vascularization, creating a favorable microenvironment for stem cell engraftment (26, 27). Each 1 × 10^6^ increase in stem cell dose reduced treatment failure risk by 18.2%, as adequate cell density is crucial for paracrine efficacy; clinical data indicate that doses < 5 × 10^6^ significantly reduce fat graft survival (28–30). Injection frequency had the most pronounced protective effect—each additional injection reduced failure risk by 42%, likely due to sustained stem cell replenishment and prolonged cytokine release, avoiding hypoxia and apoptosis associated with single high-dose injections (31).

Notably, only minor differences in the relative strength of individual factors were observed across subgroups (e.g., pre-treatment skin score had a slightly more pronounced protective effect in the skin aging subgroup), but no contradictory associations (e.g., a factor being a risk factor in one subgroup and a protective factor in another) were found. This high consistency confirms that the core predictive factors identified by the overall model are broadly applicable to patients with alopecia areata, skin aging, or scar repair (the three common indications included in this study), and further supports the reliability of the predictive model, though external multi-center validation is still needed to verify its generalizability to more diverse populations and clinical settings.

Model comparison demonstrated that the RF model achieved superior performance (AUC: 0.798/0.787 in training/validation sets) compared to SVM (0.761/0.770) and KNN (0.723/0.708), with consistently high precision, recall, and F1-scores (training F1 = 0.822; validation F1 = 0.816), indicating robust stability and generalizability. The RF algorithm’s strength lies in its ensemble learning approach, which minimizes overfitting via decision tree voting, making it ideal for complex, multifactorial datasets.

This study provides a practical tool—a nomogram—that translates the predictive model into an intuitive, points-based system for estimating individual patient risk. Clinicians can use this nomogram during preoperative consultation to stratify patients, for instance, defining those with a predicted probability of ineffectiveness ≥ 60% as high-risk. For such individuals, we recommend optimizing treatment protocols by using low-passage stem cells (≤ 3 passages), increasing injection frequency (e.g., 4–5 sessions), and considering adjunct therapies such as platelet-rich plasma. Conversely, for low-risk patients (e.g., predicted risk < 30%), standard protocols may suffice. For instance, a 44-year-old patient (≈48 points) with a disease duration of 14 months (≈42 points), diabetes (68 points), a pre-treatment skin score of 60 (≈50 points), a stem cell dose of 5 × 10^6^ (≈55 points), passage number > 3 (≈70 points), and 2 injections (≈52 points) would have a total point of ≈385, corresponding to an estimated risk of ineffectiveness of ≈0.90 (90%). This indicates a high-risk patient who may require treatment optimization (e.g., using low-passage stem cells, increasing injection frequency, or adding PRP). It should be emphasized that these treatment adjustments are currently hypothetical and have not been prospectively validated, serving as hypothesis-generating for future clinical trials. An online calculator is under development to further simplify risk estimation and support real-time clinical decision-making in practice.

However, our study has some limitations. Firstly, the single-center retrospective design may introduce selection bias, and the cohort (e.g., 29.4% with alopecia areata) cannot fully represent diverse patient populations, so multicenter validation is necessary to improve the model’s generalizability. Additionally, the model may encode site-specific practice patterns (e.g., injection frequency, dose ranges, passage thresholds) rather than solely patient biology, which could limit applicability in settings with different protocols. Secondly, due to inherent constraints of retrospective data collection, variable definitions are oversimplified: “smoking history,” “drinking history,” and “comorbidities” were only recorded as binary data (without quantitative details such as smoking duration or comorbidity severity), and “passage number” was dichotomized as ≤ 3 vs. > 3. Moreover, high collinearity between “stem cell dose” and “concentration” required excluding the latter, further reducing the granularity of predictive factors. Notably, therapeutic efficacy was defined as a binary outcome (≥ 50% improvement vs. < 50% improvement) because continuous follow-up data (e.g., precise wrinkle reduction percentage, exact hair density increase) was unavailable, which may overlook clinically meaningful mild improvements (30–49%). Additionally, collapsing three distinct clinical endpoints (hair density, wrinkle reduction, scar pliability) into a single binary label may introduce noise and ceiling/floor effects between subgroups, potentially contributing to the moderate discrimination (AUC: 0.798/0.787 in training/validation sets) of the model. Thirdly, the absence of external validation or prospective testing is a significant constraint. While internal validation showed promising results, the performance and robustness of our model have not been confirmed in an independent, external cohort or in a real-world prospective setting. Fourthly, the limited follow-up period of six months precludes the assessment of long-term outcomes and the durability of our predictions. A longer observation window would be necessary to evaluate the model’s performance over time and its impact on chronic disease progression. Finally, key molecular markers (e.g., CD90, CD105, genetic polymorphisms) that could enhance predictive accuracy were not included, leaving room for further optimization. Future prospective studies should address these gaps by collecting quantitative variable data, adopting continuous efficacy assessment tools, supplementing calibration metrics and translation tools, and incorporating molecular markers and patient-reported outcomes [e.g., Dermatology Life Quality Index (DLQI)].

In conclusion, this study established a predictive model identifying age, disease duration, diabetes, and passage number as critical determinants of therapeutic efficacy, while demonstrating the clinical applicability of machine learning for personalized treatment strategies. Preliminary data from our RF model suggest that high-risk patients might potentially benefit from low-passage stem cells (< 3 passages), increased injection frequency (4–5 sessions), and adjunct therapies (e.g., platelet-rich plasma). However, these strategies lack direct prospective validation and should be regarded as hypothesis-generating for future clinical trials.

## Data Availability

The original contributions presented in this study are included in this article/Supplementary material, further inquiries can be directed to the corresponding author.
