# Supplementary material for: A machine learning-based predictive model for stem cell therapy outcomes in plastic surgery
Source: Front Med (Lausanne). 2025 Dec 17;12:1683758. doi: 10.3389/fmed.2025.1683758 (PMC12753976; doi:10.3389/fmed.2025.1683758)
Supplement: Supplementary file 1 [file Table_1.docx]

**Supplementary Table 1.** Variable assignment table

| Variable | Meaning | Assignment |
| --- | --- | --- |
| X1 | Age | Continuous |
| X2 | Disease duration | Continuous |
| X3 | History of diabetes | 1 = Yes, 0 =No |
| X4 | Pre-treatment skin score | Continuous |
| X5 | Stem cell dose | Continuous |
| X6 | Passage number | 1=“＞3”，0=“≤3” |
| X7 | Number of injections | Continuous |
| Y | Efficacy of stem cell therapy | 1 = Ineffective, 0 = Effective |
