# Supplementary material for: A machine learning-based predictive model for stem cell therapy outcomes in plastic surgery
Source: Front Med (Lausanne). 2025 Dec 17;12:1683758. doi: 10.3389/fmed.2025.1683758 (PMC12753976; doi:10.3389/fmed.2025.1683758)
Supplement: Supplementary file 2 [file Data_Sheet_1.docx]

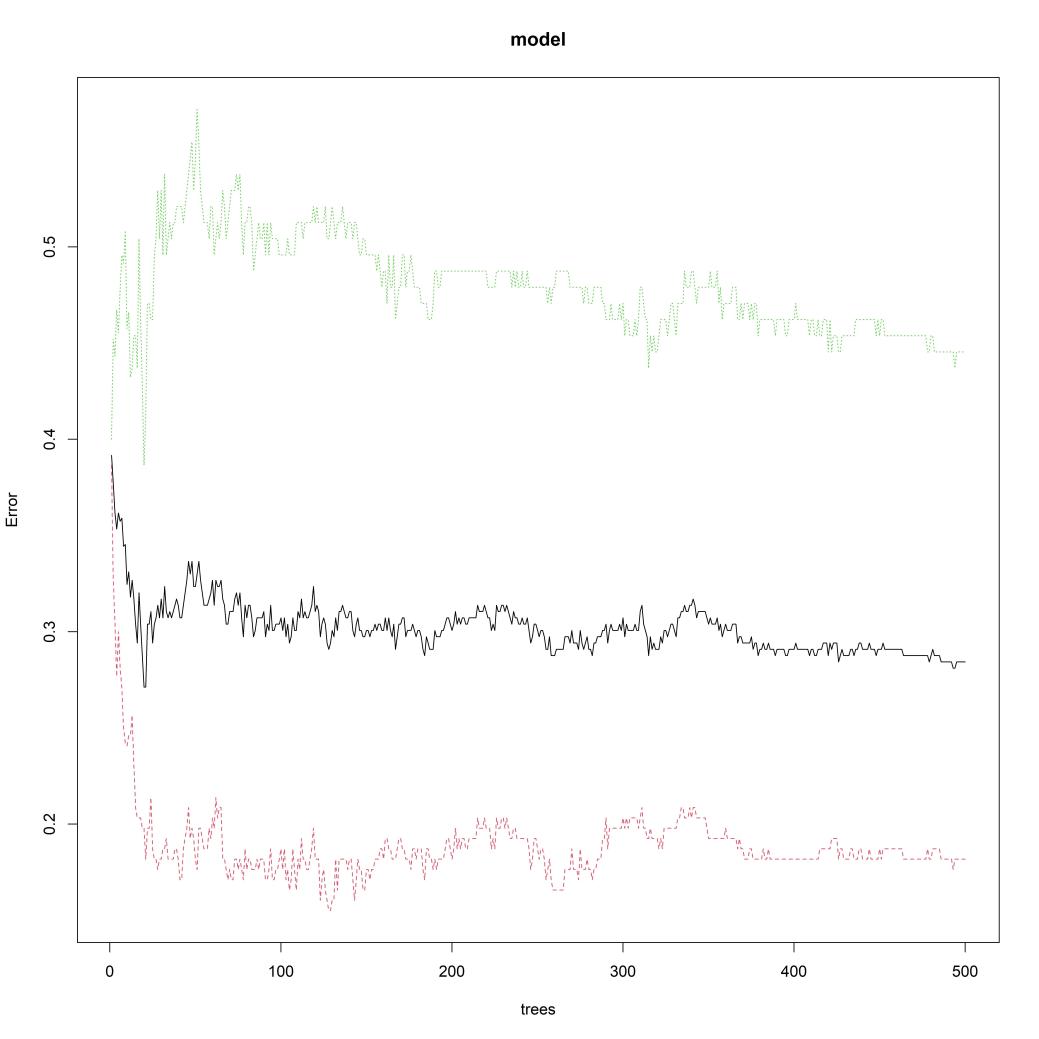


**Supplementary Figure 1.** The trend of average out of bag estimation error rate changing with the number of decision trees


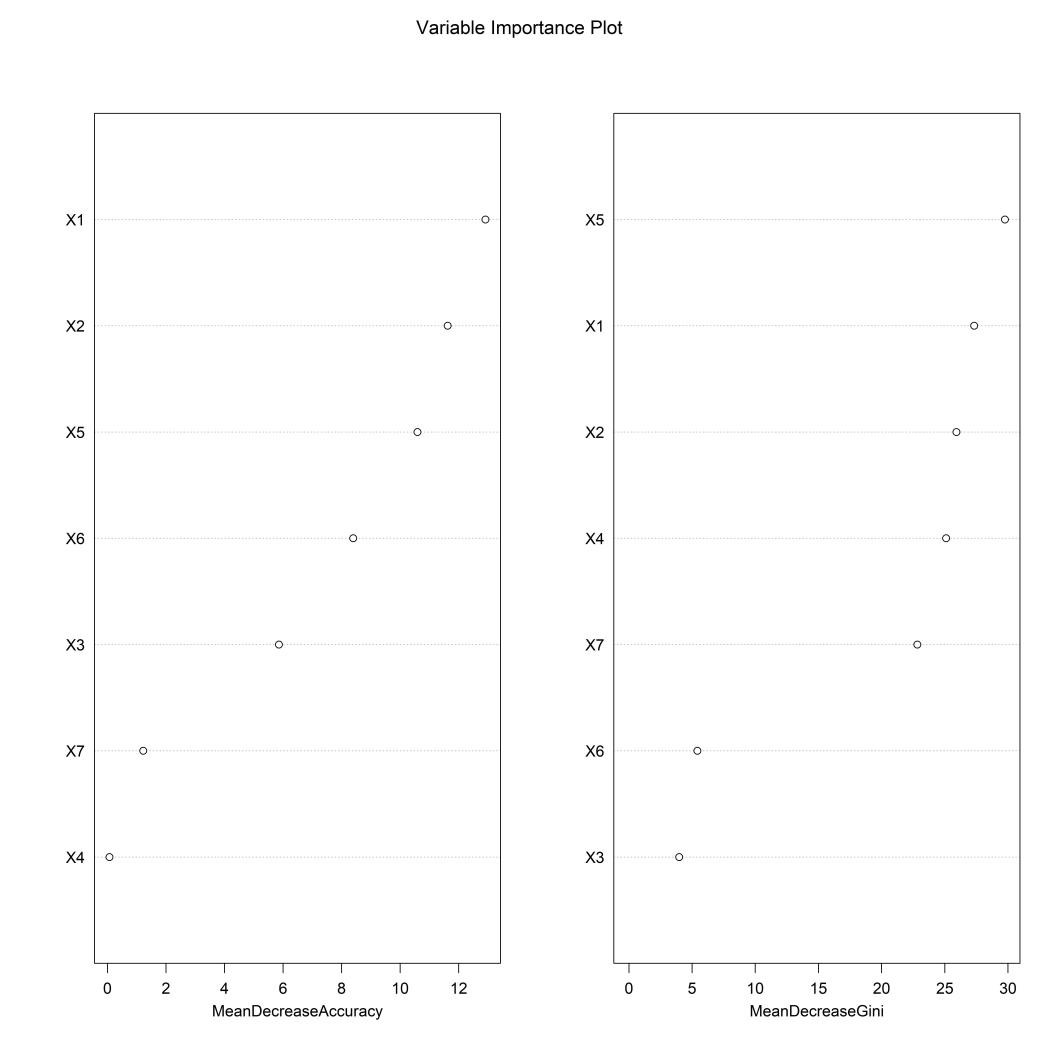


**Supplementary Figure 2.** Variable importance ranking in the random forest model (X1: Age, X2: Disease duration, X3: History of diabetes, X4: Pre-treatment skin score, X5: Stem cell dose, X6: Passage number, X7: Number of injections)
